# A New Curve of Critical Nitrogen Concentration Based on Spike Dry Matter for Winter Wheat in Eastern China

**DOI:** 10.1371/journal.pone.0164545

**Published:** 2016-10-12

**Authors:** Ben Zhao, Syed Tahir Ata-UI-Karim, Xia Yao, YongChao Tian, WeiXing Cao, Yan Zhu, XiaoJun Liu

**Affiliations:** 1 National Engineering and Technology Center for Information Agriculture, Jiangsu Key Laboratory for Information Agriculture, Jiangsu Collaborative Innovation Center for Modern Crop Production, Nanjing Agricultural University, Nanjing, Jiangsu, China; 2 Key Laboratory of Crop Water Use and Regulation, Ministry of Agriculture, Farmland Irrigation Research Institute, Chinese Academy of Agricultural Sciences, Xinxiang, Henan, China; University of Vigo, SPAIN

## Abstract

Diagnosing the status of crop nitrogen (N) helps to optimize crop yield, improve N use efficiency, and reduce the risk of environmental pollution. The objectives of the present study were to develop a critical N (N_c_) dilution curve for winter wheat (based on spike dry matter [SDM] during the reproductive growth period), to compare this curve with the existing N_c_ dilution curve (based on plant dry matter [DM] of winter wheat), and to explore its ability to reliably estimate the N status of winter wheat. Four field experiments, using varied N fertilizer rates (0–375 kg ha^-1^) and six cultivars (Yangmai16, Ningmai13, Ningmai9, Aikang58, Yangmai12, Huaimai 17), were conducted in the Jiangsu province of eastern China. Twenty plants from each plot were sampled to determine the SDM and spike N concentration (SNC) during the reproductive growth period. The spike N_c_ curve was described by N_c_ = 2.85×SDM^-0.17^, with SDM ranging from 0.752 to 7.233 t ha^-1^. The newly developed curve was lower than the N_c_ curve based on plant DM. The N nutrition index (NNI) for spike dry matter ranged from 0.62 to 1.1 during the reproductive growth period across the seasons. Relative yield (RY) increased with increasing NNI; however, when NNI was greater than 0.96, RY plateaued and remained stable. The spike N_c_ dilution curve can be used to correctly identify the N nutrition status of winter wheat to support N management during the reproductive growth period for winter wheat in eastern China.

## Introduction

Nitrogen (N) is a key input element in agriculture that increases yield. In 2014 more than 500 thousand tons of N fertilizer was applied in eastern China to satisfy agricultural production [1]. In this region, winter wheat and rice are rotated intensively to satisfy the demand of human consumption [2–3]. The average rates of N fertilizer application on winter wheat in this region are 250 kg ha^-1^, with 70% of this amount used as a basal fertilizer [4]. Due to the poor uptake capacity of N in the root system during the early growth period of winter wheat, more than 50% of N fertilizer is lost in the form of atmospheric emissions (toxic ammonia, N oxide) and leaching (nitrate ions, ammonium ions), which can cause environmental damage and decrease the efficiency of N use [5–6]. In eastern China, N use efficiency for the winter wheat is as low as about 30%, which was well lower than N use efficiency (50~60%) of the developed countries (France and Germany) [7–9]. Therefore, precise management of N fertilizer during different growth stages of winter wheat has become a core area of research; it is critical for improving N use efficiency and reducing environmental damage in eastern China.

Accurate diagnosis of crop N status is essential for precise N fertilizer management [10]. Several diagnostic tools, such as chlorophyll meters [11,12], remote sensing [13], stem sap nitrate concentration [14], and critical N (N_c_) dilution concentration [15], have been used to assess the optimal N fertilizer management to balance the sustainability and profitability of crop production. These tools differ in scope, reference spatial scales, and monetary and time resources, as well as skills and expertise required for their implementation in the field [15]. Although a chlorophyll meter is a simple way to detect crop N status, its measurements are easily affected by leaf thickness and environment [11]. Remote sensing is limited to the detection of crop N concentration under non-N-limiting treatments, because the spectral reflectance is subject to saturation under non-N-limiting conditions [13]. Stem sap nitrate concentration is influenced by cultivar, environment, and growth stage of crop [14]. In the last 30 years, an alternative technique has been developed, based on the concept of N_c_ dilution concentration in the crop, which is defined as the minimum N concentration required for maximum crop growth [15]. This method reveals the precise connection between crop growth and N absorption, which can accurately distinguish the concentration of crop N that can satisfy the demand of crop growth.

The N_c_ dilution curve was firstly developed by Lemaire and Salette [16] for tall fescue (*Festuca arundinacea*) and was established by the following power function:

$$N_{c}=a\times DM^{-b}$$ (1)

where DM is the plant dry matter (DM) expressed in t ha^-1^, *a* and *b* represent the parameters of this curve, and N_c_ is the critical N concentration of crop expressed in % DM. The N_c_ dilution curves of different crops, such as winter wheat [17, 18], rice [19], maize [20], and winter oilseed rape [21], have been generated. These curves have been successfully applied for crop N diagnoses. However, some studies found that, in recent years, the N_c_ curves of a crop have varied among different regions, genotypes and managements [17, 22, 23]. This phenomenon may be caused by DM partitioning among different plant parts (leaf, stem, and spike). Stress responses may affect the partitioning of DM between different plant organs [24] and change the shape of the N_c_ dilution curve; therefore, the N_c_ dilution curve, based on the plant DM, may not always be the most appropriate method to diagnose the status of crop N.

The theory of N_c_ dilution has also been applied to develop N_c_ curves on specific crop organs (*e*.*g*. leaf or stem), based on DM; the shape of the curves for the DM of specific parts is the similar to that of DM of entire plants [25, 26]. However, the leaf N_c_ dilution curve is easily affected by leaf aging, the increasing proportion of structural leaf tissue, and progressive shading by newer leaves [27]. Furthermore, the N_c_ curves based on leaf or stem alone were established during the vegetative growth period of the crop; thus, they cannot be used to diagnose crop N status during the reproductive period. These reasons can limit the application of these curves. During the reproductive stage, the spike of winter wheat, which is the important organ for crop yield and quality, has the highest growth. The second peak of crop N absorption is observed during this stage, as the increase of crop yield and quality is associated with an increase of N uptake in the spike [28]. Arduini et al [29] showed that the N accumulation rate of durum wheat was between 33.8 and 86.3 kg ha^-1^ at the reproductive period, which could account between 22.7% and 45.4% N accumulation rate of durum wheat. Based on the dynamic change of spike dry matter (SDM) and spike N concentration (SNC) for different crops (wheat and maize), the N_c_ concentration exists in the spikes [30, 31, 32]. The partitioning of DM and N nutrition among different plant organs is first optimized to satisfy the growth demand of the spike during the reproductive period. Under these circumstances, the shape of the spike N_c_ curve is stable.

In eastern China, winter wheat is often affected by high temperature and dry hot air during the reproductive growth period, which in turn result in premature ripening of winter wheat. The winter wheat grown under such environmental conditions suffer lower canopy photosynthesis, altered plant N metabolism [33] and shriveled grain with less grain weight during the reproductive period of winter wheat. To overcome this issue, postponing N application or foliar N application during the reproductive period of winter wheat are generally practiced in the field to increase the canopy photosynthesis capacity and recovery N metabolism process in the eastern China [34]. However, the inappropriate management of N fertilizer has an adverse effect on the spike growth of winter wheat during this period. For estimating the appropriate N fertilizer requirements, it is essential to develop a new N_c_ curve based on SDM in eastern China, which can be used to diagnose the N status of winter wheat during the reproductive growth period.

The objectives of this study were to develop and validate a N_c_ dilution curve based on SDM, to compare this curve with the existing N_c_ dilution curves of winter wheat, and to assess the plausibility and accuracy of this curve for estimating N status of winter wheat in eastern China.

## Materials and Methods

### Ethics statement

The Experimental plots are located in land owned and managed by Nanjing Agricultural University, Nanjing, China. Nanjing Agricultural University permits and approvals obtained for the work and study. The field studies did not involve wildlife or any endangered or protected species.

### Experimental design

**Fig 1:**
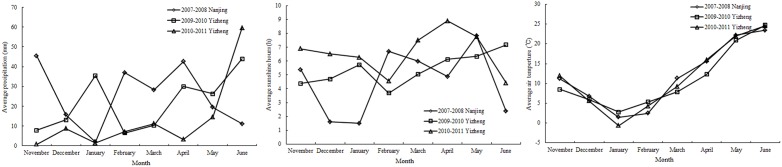
Average monthly precipitation (mm), sunshine hours (h), air temperature (°C) during the three season period.

Data were obtained from four field experiments, conducted at different sites and in different years, that included varied N rates (0–375 kg ha^-1^) and six wheat cultivars (Aikang58, Huaimai17, Ningmai9, Ningmai13, Yangmai12, and Yingmai16), as summarized in Table 1. The wheat cultivars were bought in the market. Experiments 1 and 2 involved seven N treatments (0–270 kg ha^-1^) and four cultivars (Ningmai9, Aikang58, Yangmai12, Huaimai17) in a randomized block design with three replications. Experiments 3 and 4 involved six N treatments (0–375 kg ha^-1^) and two cultivars (Yangmai16 and Ningmai13) in a randomized block design with three replications. N fertilizer was applied before sowing (50%) and at the jointing stage (50%). At Nanjing (32°04′N, 118°78′E, Experiments 1 and 2), winter wheat was planted on 5 November 2007 at a density of 150 × 10^4^ ha^-1^, with rows 25 cm apart. At Yizheng (32°27′N, 119°16′E, Experiments 3 and 4), winter wheat was planted on 5 November 2009 and 7 November 2010, at a planting density of 180 × 10^4^ ha^-1^, with rows 25 cm apart. The weather conditions of the experimental sites during the three seasons were shown in Fig 1. The amounts of P and K fertilizers applied were based on soil test recommendations to satisfy the requirements of plant growth. Crop water requirements were satisfied by irrigation, weeds were controlled by hand, and pests and diseases were completely controlled by chemical application.

**Table 1:** The basic information about the four experiments conducted in present study.

	Season	Soil characteristics	Cultivar	N rate (kg N ha^-1^)	Sampling period	Cropping system	Experiment classification
Experiment 1	2007/2008	Type: sandy soil^#^	Ningmai9 (NM9)	0 (N0)	Anthesis	Rice-wheat	Validation
(Nanjing)		Organic matter^†^:16.46 g kg^-1^		90 (N1)	Filling stage		
		Total N^‡^: 1.3 g kg^-1^		180 (N2)			
		Available P^¶^: 54 mg kg^-1^		270 (N3)			
		Available K^§^: 90.5 mg kg^-1^					
Experiment 2	2007/2008	Type: sandy soil	Aikang58 (AK58)	75 (N1)	Anthesis	Rice-wheat	Validation
(Nanjing)		Organic matter:10.43 g kg-1	Yangmai12 (YM12)	150 (N2)	Filling stage		
		Total N: 0.9 g kg^-1^	Huaimai17 (HM17)	225 (N3)			
		Available P: 13.94 mg kg^-1^					
		Available K: 151 mg kg^-1^					
Experiment 3	2009/2010	Type: clay soil	Ningmai13 (NM13)	0 (N0)	Anthesis	Rice-wheat	Construction
(Yizheng)		Organic matter: 18.9 g kg^-1^	Yangmai16 (YM16)	75 (N1)	Filling stage		
		Total N: 1.5 g kg^-1^		150 (N2)			
		Available P: 34 mg kg^-1^		225 (N3)			
		Available K: 90 mg kg^-1^		300 (N4)			
Experiment 4	2010/2011	Type: clay soil	Ningmai13 (NM13)	0 (N0)	Anthesis	Rice-wheat	Construction
(Yizheng)		Organic matter: 13.5 g kg^-1^	Yangmai16 (YM16)	75 (N1)	Filling stage		
		Total N: 1.1 g kg^-1^		150 (N2)			
		Available P: 43 mg kg^-1^		225 (N3)			
		Available K: 82 mg kg^-1^		300 (N4)			
				375 (N5)			

^#^ Soil Survey Staff. 2006. Keys to soil taxonomy. USDA. Natural Resources Conservation Service. 10th ed.

^†^ Walkley-Black titration method

^‡^ Dry combustion

^¶^ 0.05 M HCl

^§^ 1 M NH_4_OAc

Data from experiments (3 and 4) conducted during 2009–2011 seasons were used to develop the N_c_ curve, while the data acquired from experiments (1 and 2) conducted in 2007–2008 season were used to test the N_c_ curve (Table 1, very informative).

### Sampling and measurement

During the reproductive growth phase, 20 plants per plot were harvested to determine SDM and SNC. Wheat spikes were oven-dried at 105°C for half an hour to rapidly stop metabolism and then at 80°C until they reached a constant weight to obtain SDM. Dried spikes were ground in a sample mill, passed through a 1-mm sieve, and stored in plastic bags for chemical analysis. SNC was determined by the micro-Kjeldahl method [35].

### Data analysis

Identifying the N_c_ point at which N fertilizer neither limits plant growth nor enhances it is essential for constructing a spike N_c_ dilution curve. The experimental data from 2009–2011 seasons were used to determine the spike N_c_ dilution curve (Table 1). The N-limiting treatment was defined as a treatment in which additional N fertilizer application led to a significant increase in the SDM. The non-N-limiting treatment was defined as a treatment in which N fertilizer application did not lead to an increase in the SDM but resulted in a significant increase in the SNC [17]. The SDM and SNC under different applied N rates were compared by using the least significant difference test (LSD) at the 95% level of significance (SPSS Inc., Chicago. IL, USA). For each sampling period, a critical point was defined as follows: (a) the data of SDM and SNC was used to identify if N treatment limited the growth of the crop; (b) a simple linear regression was used to fit data from the N-limiting treatments (the oblique line); (c) The maximum SDM was calculated with data from the non-N-limiting treatments as the average of the observed data (the vertical line); (d) the N_c_ point corresponded to the ordinate of the intersection point of the oblique and vertical lines; and (e) these N_c_ points were fitted using a power regression equation to construct the spike N_c_ curve.

Data points from non-N-limiting treatments (N4 treatment in 2009–2010, N5 treatment in 2010–2011) were used to determine the maximum N curve (N_max_). The data points from N-limiting treatments (N0 treatments in 2009–2011) were used to determine the minimum N curve (N_min_).

The N nutrition index (NNI) defined by Lemaire et al [14] was used to characterize crop N status:

$$NNI=SNC/N_{c}$$ (2)

If NNI = 1, N nutrition is considered optimal. If NNI>1, N nutrition is considered excessive, and if NNI<1, N nutrition is considered insufficient.

The relative yield (RY) was obtained by dividing the yield at a given N rate by the highest yield among all N treatments. The coefficients of determination (*R*^*2*^) for the relationship between RY and NNI were calculated using SPSS 18.0.

## Results

### Spike dry matter and spike nitrogen concentration under varied N treatments

The N application rate had a significant effect on SDM during the reproductive growth period. SDM increased gradually with the increasing rate of N application and ranged from 0.752 to 7.233 t ha^-1^ depending on the cultivar, sampling date, and season (Table 2). In 2009–2010, SDM increased significantly from N0 to N2 treatments, but there were no significant differences among N3 and N4 treatments. In 2010–2011, a similar trend was observed; however, the maximum SDM was obtained in the N3 treatment. The N rates were not significantly different among N3, N4, and N5 treatments. During each experimental season, SDM conferred with following inequality under different N treatments:

2009−2010: SDM0<SDM1<SDM2=SDM3=SDM4 (3)

2010−2011: SDM0<SDM1<SDM2<SDM3=SDM4=SDM5 (4)

where SDM0, SDM1, SDM2, SDM3, SDM4, and SDM5 are the SDM values of N0, N1, N2, N3, N4, and N5, respectively.

**Table 2:** Changes of spike dry matter (t ha^-1^) for winter wheat with time (days after sowing) under different N application rates in experiments conducted during 2009–2011 seasons.

Season	Cultivar	N treatment	Days after sowing					Boundary N curve data set
			174	181	191	201	210	
2009/2010	YM16	N0	1.128c	1.854c	2.354c	4.397c	4.938c	N_min_
		N1	1.303b	2.497b	2.997b	4.997b	5.825b	
		N2	1.495a	2.734a	3.734a	5.85a	6.957a	
		N3	1.459a	2.889a	3.389a	5.686a	7.275a	
		N4	1.459a	3.053a	3.73a	5.699a	6.847a	N_max_
								
	NM13	N0	1.595c	2.08c	2.68c	4.246c	4.887c	N_min_
		N1	1.815b	2.421b	2.921b	4.674b	5.754b	
		N2	2.032a	2.867a	3.267a	5.361a	6.854a	
		N3	2.01a	2.624a	3.224a	5.183a	7.085a	
		N4	1.978a	2.774a	3.374a	5.276a	6.974a	N_max_
								
2010/2011	YM16	N0	1.318d	2.054d	2.569d	3.54d	4.12d	N_min_
		N1	1.545c	2.362c	3.139c	4.628c	5.234c	
		N2	1.785b	2.767b	3.746b	5.26b	6.12b	
		N3	2.048a	3.264a	4.5a	6.158a	7.233a	
		N4	1.998a	3.387a	4.134a	5.985a	7.042a	
		N5	1.999a	3.278a	4.389a	5.999a	7.145a	N_max_
								
	NM13	N0	1.565d	2.045d	2.86d	3.907d	4.452d	N_min_
		N1	1.772c	2.456c	3.349c	4.615c	5.314c	
		N2	2.016b	2.847b	3.629b	5.081b	6.04b	
		N3	2.258a	3.058a	3.78a	5.827a	6.974a	
		N4	2.233a	3.124a	3.749a	5.634a	6.754a	
		N5	2.198a	3.087a	3.924a	5.735a	6.834a	N_max_

F prob statistic significant at 0.01 probability level

**Fig 2:**
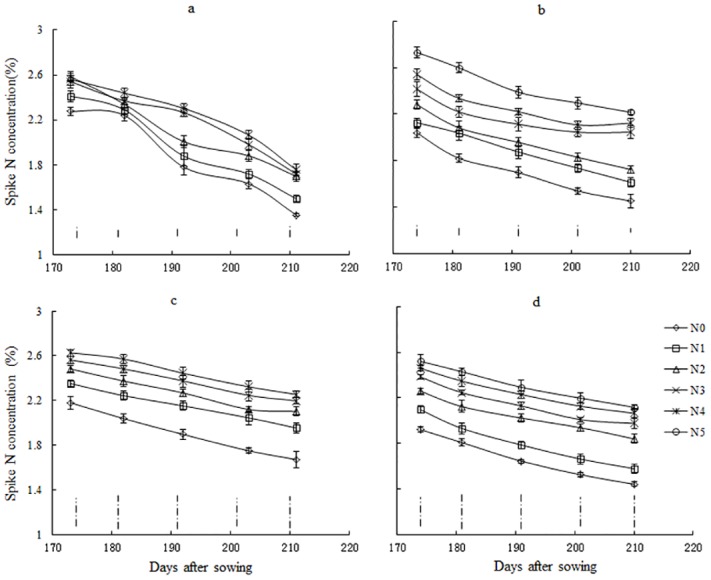
Changes in winter wheat spike N concentration with time under different N application rates in the 2009 and 2011 seasons. a: 2009–2010 NM13, b: 2010–2011 NM13, c: 2009–2010 YM16, d: 2010–2011 YM16. Data are presented as days after sowing. The vertical bars represent standard error of the mean value on each sampling date. The vertical dotted bars without line end represent LSD values (*P*<0.05) on each sampling date.

SNC declined gradually during the reproductive growth period, and a higher rate of N application generally resulted in a higher SNC. SNC ranged from 1.22% to 2.65% for NM13 (Fig 2a) and from 1.45% to 2.72% for YM16 (Fig 2c) during the 2009–2010 season. In contrast, it ranged from 1.27% to 2.43% for NM13 (Fig 2b) and from 1.04% to 2.33% for YM16 (Fig 2d) during the 2010–2011 season.

### Determining the spike critical nitrogen dilution curve for winter wheat

**Fig 3:**
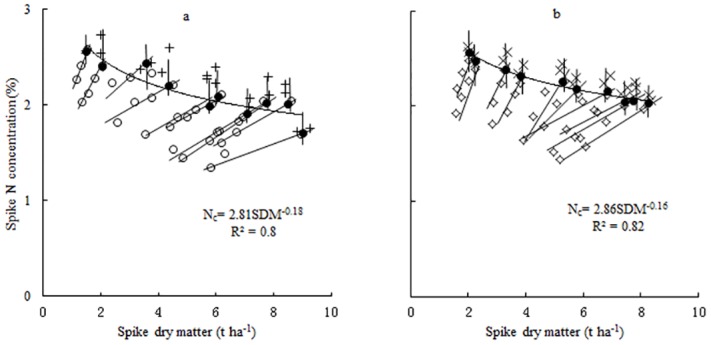
Critical N data points used to develop spike N_c_ curves. a: 2009–2011 NM13, b: 2009–2011 YM16. The symbols (◇) and (○) represent the data points obtained from the N-limiting treatments for YM16 and NM13, and (×) and (+) represent the data points obtained from the non-N-limiting treatments for YM16 and NM13. The symbols (●) represent the calculated N_c_ points for each sampling date. The solid line represents the spike N_c_ curve that describes the relationship between spike N_c_ concentration and SDM of winter wheat.

Following the computational procedures of Justes et al [17], spike N_c_ points were determined for each sampling date during the reproductive growth period for NM13 and YM16. The SDM data, ranging from 0.752 to 7.233 t ha^-1^, allowed the calculation of the N_c_ points. Spike N_c_ points were calculated as the intercepts between vertical and oblique lines for each sampling date (Fig 3). There was a decreasing trend of N_c_ points with increasing SDM of winter wheat; the trend lines could be fitted by the following negative power regression equation:

$$NM13:\;N_{c}=2.81\times SDM^{-0.18}$$ (5)

$$YM16:\;N_{c}=2.86\times SDM^{-0.16}$$ (6)

**Fig 4:**
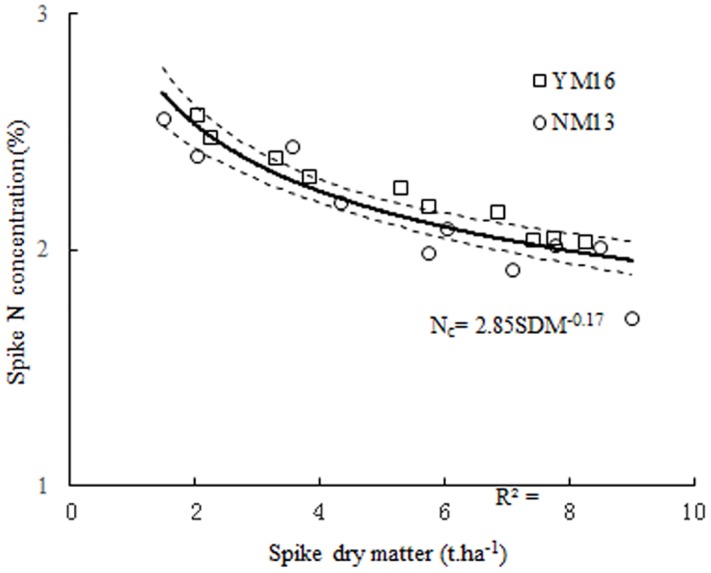
Critical N data points for spike N_c_ curve definition using pooled data from two cultivars. The solid line represents the critical N dilution curve (N_c_ = 2.85×SDM^-0.17^, *R*^*2*^ = 0.8) that describes the relationship between the N_c_ concentration and SDM of wheat. The dotted lines represent the 95% confidence interval (*P* = 0.95).

The two curves showed non-significant differences when compared according to calculation procedures recommended by Mead and Curnow [36]. Therefore, the data points of two curves were pooled to develop the following unified N_c_ curve (Fig 4):

$$N_{c}=2.85\times SDM^{-0.17}$$ (7)

**Fig 5:**
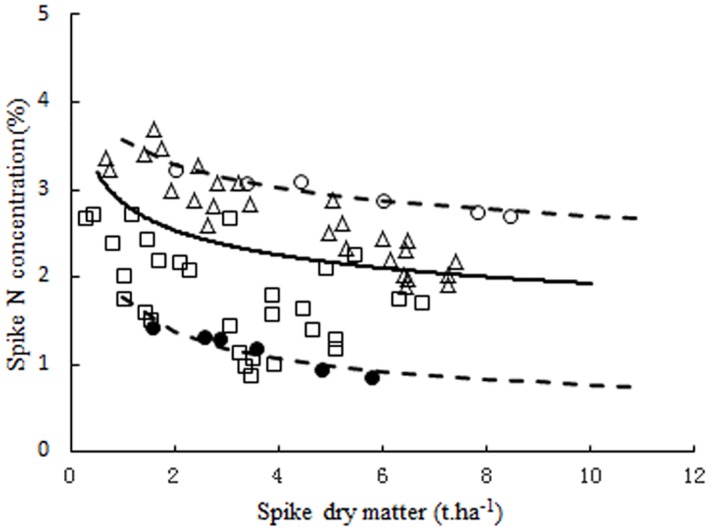
Comprehensive validation of the spike N_c_ curve using the independent data set from the experiment conducted during 2007–2008. Data points (□) and (△) represent N-limiting and non-N-limiting treatments, respectively. The solid line in the middle represents the critical N curve (N_c_ = 2.85×SDM^-0.17^) that describes the relationship between the critical N concentration and spike dry matter of rice. The data points (●) and (○) that were not used for establishing the parameters of the allometric function (2009–2011) were used to develop two limit curves (--------): minimum limit curve (N_min_ = 1.86×SDM^-0.41^) and maximum limit curve (N_max_ = 3.57×SDM^-0.12^).

The spike N_c_ curve was validated using an independent data set (N = 56) obtained from the experiments conducted in 2007–2008 (Experiment 1 and 2, S1 Table). The result showed that the newly developed curve can clearly discriminate between the N-limiting and non-N-limiting growth conditions of winter wheat during the reproductive growth period. Cultivar, season, site, and growth stage did not significantly affect this curve. The data points from N-limiting treatments were below this N_c_ curve, and the points from non-N-limiting treatments were near or above this curve (Fig 5). To determine the N_max_ curve, data points were only selected from non-N-limiting treatments (n = 6) during the 2009–2011 seasons. For the N_min_ curve, data points were selected from the treatment without N application (n = 6) during the 2009–2011 seasons. The N_max_ and N_min_ curves could represent the possible range of SNC (Fig 5).

### Change of nitrogen nutrition index under different N treatments during 2009–2011 seasons

**Fig 6:**
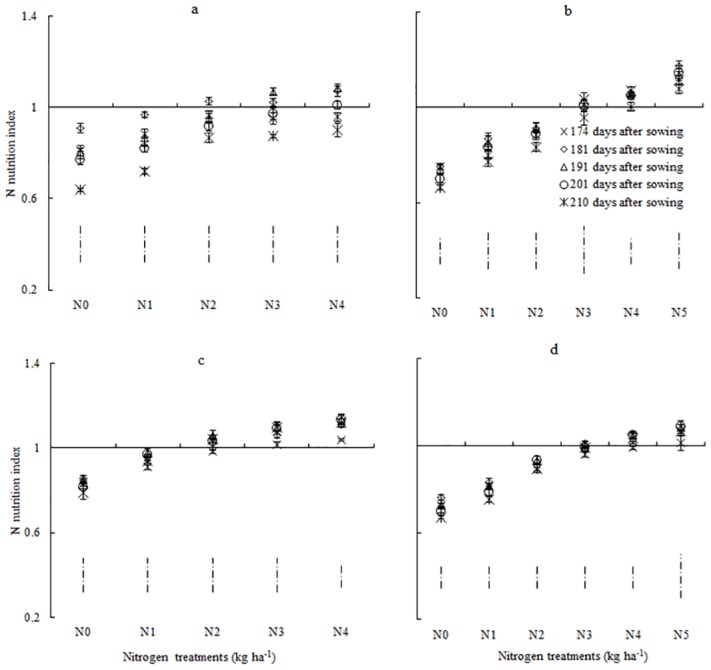
Changes in the N nutrition index for YM16 and NM13 during the reproductive growth period. a: 2009–2010 NM13, b: 2010–2011 NM13, c: 2009–2010 YM16, d: 2010–2011 YM16. The vertical bars represent standard error of the mean value on each sampling date. The vertical dotted bars without line end represent LSD values (*P*<0.05) on each sampling date.

Our results showed that NNI was significantly different across the growing seasons, N treatments, cultivars, and growth stages. After anthesis, NNI decreased with the declining rate of N application, and NNI ranged between 0.62 and 1.1 in NM13 (Fig 6a and 6b) but ranged between 0.64 and 1.1 in YM16 (Fig 6c and 6d) in 2009–2011. The NNI values were <1 for N0 and N1 in the 2009–2010 seasons and for N0, N1, and N2 in the 2010–2011 season (N-limiting treatments). The minimum NNI values were observed at the filling stage. The NNI values of N2 treatments in the 2009–2010 season and those of N3 treatments during the 2010–2011 seasons were close to 1; this indicates that the N rate was optimal for winter wheat growth. The NNI values were >1 for N3 and N4 treatments during the 2009–2010 season and for N4 and N5 treatments during the 2010–2011 season; this indicates the presence of surplus N uptake in spikes of winter wheat. These results illustrate that NNI can be used as a robust diagnostic tool for N status of winter wheat under different N growing conditions.

**Fig 7:**
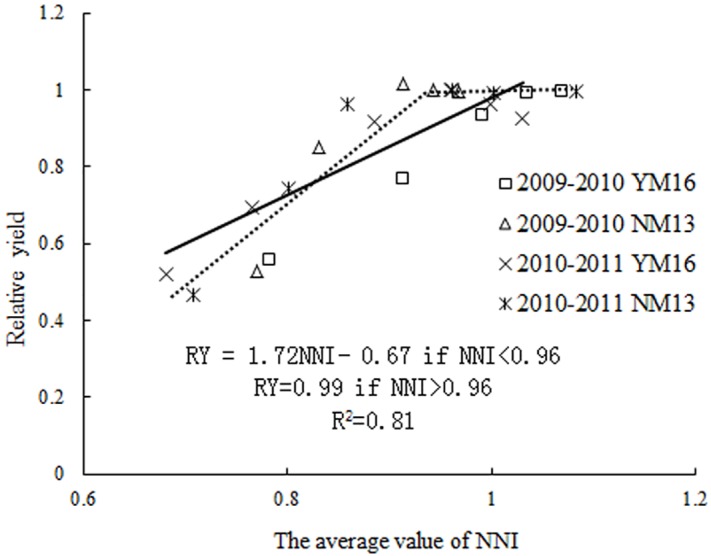
The relationship between RY and NNI for winter wheat in 2009–2011. The NNI value was averaged over all sampling dates.

Further, the relationship between RY and NNI was constructed with a linear-plateau function. As seen in Fig 7, the RY was near 1.0 for NNI values > 0.96. When NNI was below 0.96, the RY decreased.

## Discussion

Winter wheat, an important cereal crop in China, existed with the unreasonably large application of N fertilizer for a long time. Excessive N fertilizer cannot ensure a significant increase in crop productivity, yet its abundant application can induce a decrease in crop yield and cause environmental damage. Efficient use of N fertilizer is required to optimize different crop production systems. In recent years, there has been an increasing demand for simple, accurate, and stable tools for diagnosing the status of crop N, which can provide appropriate on-farm N management. With the advancement of N dilution principle, the diagnosis model based on this theory has been developed and used to guide agricultural field production. So far, there have been some studies about crop N_c_ curves, based on plant DM during the vegetative period, but no attempts have been developed to a N_c_ curve on the basis of SDM during the reproductive period. This study supports the idea of the current N_c_ dilution phenomenon in the spike of winter wheat. Through field experiments in three seasons in eastern China, we have constructed a N_c_ dilution curve based on SDM and provided a new way of diagnosing and regulating the N status of winter wheat during the reproductive period.

### Variability of spike nitrogen concentration in winter wheat

A decreasing trend of SNC was observed with increasing SDM during the reproductive growth period. This trend was due to the change in the proportion of grain/glume during spike growth. There was a significant variation in SNC in the growth of winter wheat, even when SDM had a constant value (Fig 5). Two boundary curves of maximum and minimum N have been developed to characterize the dynamic range of SNC (Fig 5). The equations for N_max_ and N_min_ curves were:

$$N_{max}=3.57\times SDM^{-0.12}$$ (8)

$$N_{min}=\;1.86\times SDM^{-0.41}$$ (9)

The N_max_ curve can be seen as the first assessment of a maximum spike N dilution curve. This curve was obtained under non-N-limiting treatments, where the accumulated rate of the SDM and N nutrition was the most adequate. This curve represents the maximum N uptake capacity of the spike, which is regulated by mechanisms directly associated with growth or indirectly *via* N metabolism [37]. The N_max_ curve showed that there was an excessive uptake of N in N5 treatment during the 2010–2011 seasons. In addition, SDM did not increase with increasing N rate. When SNC was between the spike N_max_ curves and the spike N_c_ dilution curve, the rate of spike N uptake was regulated by mineral N availability in the soil and N transfer capacity of other plant organs (*e*.*g*. stem or leaf) during the reproductive period. Notably, this was independent of spike growth rate. When SNC was below this N_c_ curve, N uptake was controlled by available mineral N in the soil, which in turn determined the growth rate of the spike [38, 39]. The N_min_ curve was considered the lowest boundary where the N metabolism would cease to function [40]. It reflects the minimum N uptake of spike under N0 treatments in this study, which was used as the threshold concentration of proper metabolic functionality of the spike.

Parameter *b* (0.4) of the N_min_ curve was well above parameter *b* (0.12) for the N_max_ curve; this indicates that extent of N dilution in the N_min_ curve was stronger than that in the N_max_ curve. In N-limiting treatments, spike N uptake cannot satisfy the demand of spike growth, due to deficient available N in the soil, which causes a rapid decrease of SNC. However, soil N can satisfy the growth demand of spike under the non-N-limiting treatments during the reproductive period, inducing a slower decrease of SNC in relation to the N_min_ curve.

### Comparison with other critical nitrogen dilution curves

**Fig 8:**
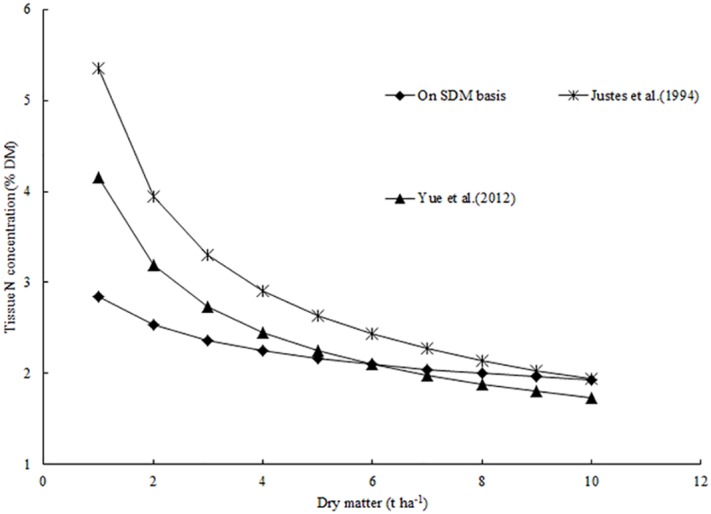
Comparison of different N_c_ curves of winter wheat. The symbol * represents the N_c_ curve of winter wheat (N_c_ = 5.35×DM^-0.4^) based on plant DM in France. The symbol ▲ represents the N_c_ curve of winter wheat (N_c_ = 4.15×DM^-0.38^) based on plant DM in the North China Plain. The symbol ◆ represents the N_c_ curve (N_c_ = 2.85×SDM^-0.17^) based on SDM in eastern China.

The N_c_ dilution curve, based on SDM, was N_c_ = 2.85×SDM^-0.17^ and was lower than the N_c_ dilution curve on the basis of plant DM (Fig 8). Parameter *a* represented the N_c_ of spike when SDM was 1 t ha^-1^. The value of parameter *a* (2.85) was smaller than the value of parameter *a* (5.35) for the N_c_ curve based on plant DM [17]. The discrepancy was related to differences in circumstance, growth stage, and cultivar. Firstly, the growth environment of winter wheat induces the differences between these curves. Justes et al [17] compared the N_c_ dilution curve of winter wheat developed in different locations (France, Belgium, and Sweden); the result showed that the N_c_ dilution curve in France was similar with that in Belgium, but the curve in France was higher than that in Sweden across a large range of plant DM under different environmental conditions. In present study, the data acquired from the multi-locational experiments conducted at in the same region (eastern China) was used to develop and validate the spike N_c_ dilution curve (Table 1), which was lower than the reference curve of Justes et al [17]. The growing environment of winter wheat was similar at all the three locations in the region. Eastern China is categorized by a subtropical-temperate climate with cold winter and hot summer. However, the climate type of experiment conducted at different locations in northern France was temperate maritime climate, with cold summer and the warmer winter. The climatic conditions of northern France are more suitable for growing of winter wheat due to the longer growth period of winter wheat in northern France as compared to that in eastern China [17, 41]. For example, the beginning of anthesis was after 125 days of 1 January in eastern China, but in northern France it was 144 days after 1 January. The shorter growth period in eastern China indicated that winter wheat do not have more opportunities to absorb N from the soil [41]. Secondly, grain N concentration of winter wheat in France was higher than that in China. Cui et al [42] have reported that the grain N concentration of wheat cultivars in China was 2.1–2.3%; however, they were 2.2–2.7% in Europe [43]. Thirdly, the development stage of winter wheat when SDM was 1 t ha^-1^ is later than when plant DM was 1 t ha^-1^, the extent of N dilution in the late growth stage was more obvious than in the earlier growth stage. In conclusion, the above three reasons led to a lower N_c_ based on SDM than that based on plant DM (Fig 8), and the spike N_c_ dilution curve may be more suitable to diagnose the N nutrition status of winter wheat in the eastern China.

Parameter *b* represents the decreasing level of SNC during crop growth and was dependent on spike N uptake relative to SDM accumulation. The decrease in SNC is attributed to two processes: (i) a large photosynthetic product of other vegetative parts (stem and leaf), transferred to grain for yield formation during the reproductive growth period [44], which causes the faster rate of SDM accumulation; (ii) the N nutrition of glume and stalk was transferred to grain for protein formation, which results in a declining N concentration of glume and stalk during the reproductive growth period.

The difference between the values of parameter *b* (0.17) in present study and that of (0.44) the curve developed by Justes et al [17] indicated that the level of spike N dilution was not as marked as the level of plant N dilution. These differences were associated with the transfer of large amounts of N to the spike to satisfy yield and proteinformation and relatively slower decline of SNC in the spike because the spike was theepicenter of plant growth during the reproductive period [45]. Second, during the vegetative stage, the proportion of structural DM (stem) with low N concentration increased, accompanying a decline in the proportion of functional DM (leaf) with high N concentration [46], and plant N concentration decreased more obviously than SNC.

### Application of the critical nitrogen dilution curve to diagnose crop nitrogen status and growth model

The N_c_ dilution curve was developed on the basis of actual crop growth, which had explicit biological significance. Firstly, this curve can be used to diagnose the N status of winter wheat. Previously, N_c_ curves of winter wheat were established during the vegetative growth period [17, 41]; however, the second peak of N uptake was from anthesis to filling stage for winter wheat [28], so it is essential to develop a curve for diagnosing the N status of winter wheat after anthesis. The N_c_ curve based on SDM was validated with an independent data set in the present study; our results indicated that this curve can accurately distinguish N-limiting and non-N-limiting treatments during the reproductive growth period of winter wheat. It is very appealing to use this N_c_ curve as a diagnosis tool for improving cost-effective and environmentally ideal N fertilization. For this purpose, the NNI value was calculated on the basis of this N_c_ curve. This value can be used to signify the N status of winter wheat quantitatively, and classify whether N is deficient, optimum, or excessive. The value of NNI in the spike varied from 0.62 to 1.1 and was affected by N treatments, growth stage, and seasons. Similar values for NNI in the plant have already been reported for crops like rice [19], corn [20] and cotton [22]. However, NNI has limitations as a diagnosis tool because the determination of NNI is time consuming and requires destructive sampling, which can be similarly estimated by non-destructive methods (Remote sensing, Chlorophyll meter). There was a good relationship between the measured value of these tools and NNI [47–48]. These indirect methods could possibly be a substitute for assessing NNI under different environments.

Secondly, the N_c_ dilution curve can be integrated into the crop growth models, which may predict the dynamic change of spike N uptake and transfer. Reyes et al [49] indicated that the N_c_ dilution curve may better inform N uptake and cycling within process-based models of grasslands. Stockle and Debaeke [50] compared the simulation effect of four growth models (AFRCWHEAT2, Daisy, EPIC, and CropSyst) to estimate the N requirement of winter wheat. The result indicated that the N_c_ dilution curve simulated in the CropSyst model can precisely calculate crop N requirement on a daily basis. In contrast, the other models, which were based on empirical relationships, were less accurate at simulating crop N requirement. In the CERES-wheat model, an N factor was used to calculate the effect of N on the SDM accumulation or spike N uptake in different circumstances. This factor was determined by a plant N_c_ curve as a function of developmental stage based on an empirical relationship; Ritchie et al [51] has reported that the N_c_ curve based on empirical relationships fails to characterize N_c_ concentrations, especially when crop development is increasing rapidly. Predictions of SDM accumulation, spike N uptake and transfer may be not accurate and rational by means of plant N_c_ curve; thus, it is possible to use the spike N_c_ curve instead of the plant N_c_ curve to simulate the effect of N on spike growth and development.

## Conclusion

The present study confirmed the correlated decrease in SNC with the increase in SDM, with a higher N application rate generally increasing SNC. Based on the theory of N_c_ dilution, we developed a new N_c_ curve on the basis of SDM for winter wheat. This curve was described by the equation N_c_ = 2.85×SDM^-0.17^ for SDM, ranging from 0.752 to 7.233 t ha^-1^. This curve was lower than the curve based on plant DM for winter wheat. The curve was tested with an independent data set, indicating that spike N_c_ curve did not appear to be significantly affected by cultivar, season, site, and growth stage. The NNI values were <1 under N-limiting treatments, and >1 under non-N-limiting treatments. When the N application rate was optimum, NNI was near 1. RY increased with the increasing NNI, but when NNI>0.96, RY kept stable. We conclude that the spike N_c_ curve offers a new perspective to understand crop N status and can be adopted as a practical diagnosis tool during the reproductive growth period for effective N management in eastern China.

## Supporting Information

S1 Table The validation values for critical nitrogen curve based on spike dry matter in the study. (XLSX)
